# Investigating Predictors of Psychological Distress for Healthcare Workers in a Major Saudi COVID-19 Center

**DOI:** 10.3390/ijerph19084459

**Published:** 2022-04-07

**Authors:** Hussain Alyami, Christian U. Krägeloh, Oleg N. Medvedev, Saleh Alghamdi, Mubarak Alyami, Jamal Althagafi, Mataroria Lyndon, Andrew G. Hill

**Affiliations:** 1 College of Medicine, Taif University, P.O. Box 11099, Taif 21944, Saudi Arabia; 2 Department of Psychology and Neuroscience, Auckland University of Technology, Private Bag 92006, Auckland 1142, New Zealand; chris.krageloh@aut.ac.nz; 3 School of Psychology, University of Waikato, Private Bag 3105, Hamilton 3240, New Zealand; omedvede@waikato.ac.nz; 4 Department of Civil Engineering, College of Engineering, Taif University, P.O. Box 11099, Taif 21944, Saudi Arabia; sjalghamdi@tu.edu.sa; 5 Administration Building, King Faisal Medical Complex, Taif 26514, Saudi Arabia; mralyami@moh.gov.sa (M.A.); joalthagafi@moh.gov.sa (J.A.); 6 Centre for Medical and Health Sciences Education, The University of Auckland, Building 409, 24 Symonds Street, City Campus, Auckland 1010, New Zealand; mataroria.lyndon@auckland.ac.nz; 7 South Auckland Clinical Campus, The University of Auckland, Level 2, North Wing, Esmé Green Building 30, Middlemore Hospital, 100 Hospital Road, Otahuhu, Auckland 1062, New Zealand; a.hill@auckland.ac.nz

**Keywords:** fear, health personnel, mental health, COVID-19, depression, anxiety

## Abstract

This study investigated the relationship between fear of COVID-19, previous exposure to COVID-19, perceived vulnerability to disease, sleep quality, and psychological distress among healthcare workers (HCWs) in Taif city in Saudi Arabia, which has a population of 702,000 people. A cross-sectional study design was adopted. HCWs (*n* = 202) completed a survey containing the Fear of COVID-19 Scale (FCV-19S), Perceived Vulnerability to Disease (PVD), Pittsburgh Sleep Quality Index (PSQI), and Depression, Anxiety, and Stress Scale (DASS-21). FCV-19S and sleep quality were significant predictors for psychological distress. Female gender was a significant predictor for depression and stress. Single, divorced, and widowed marital status were predictive for anxiety. FCV-19S was weakly correlated with PVD but moderately with depression, anxiety, and stress. Of the two PVD subscales, perceived infectability was weakly correlated with psychological distress. PVD and previous experience with COVID-19 were not significant predictors. Sleep quality and FCV-19S were major predictors of psychological distress. Findings indicated that poor sleep quality was strongly associated with psychological distress, while fear of COVID-19 had a moderate association. Such results support the need to design and implement psychological programs to assist HCWs in dealing with the psychological impact of this ongoing pandemic.

## 1. Introduction

Coronavirus disease (COVID-19) was first identified in December 2019 in Wuhan, China [1]. COVID-19 was declared a public health emergency of international concern by the World Health Organization (WHO) on 30 January 2020 [2]. The COVID-19 pandemic has been described as one of the most serious pandemics Saudi Arabia and the world has faced over the last century [3]. The seriousness of this pandemic was its high potential to spread to others compared to past coronavirus types such as the Middle East respiratory syndrome coronavirus (MERS-CoV) [4]. This is measured by the number of infected individuals due to the fact of one infected case, which was 2.5 for COVID-19 compared to 0.9 for MERS-CoV [4].

Healthcare workers (HCWs) represent the first line in the fight against the pandemic [5]. This has come with significant effects on psychological well-being in terms of depression, anxiety, or stress [6]. Recent local and international studies have shown that many HCWs already experience depressive and anxiety-related symptoms as well as insomnia [3,7,8,9,10,11,12,13,14,15,16,17].

Internationally, a recent meta-analysis involving 97,333 HCWs, published in 2021, showed that HCWs’ depression and anxiety pooled prevalence during COVID-19 was 21.7% and 22.1%, respectively [4]. This meta-analysis found that the highest pooled prevalence of these conditions was in Middle Eastern studies (34.6% for depression and 28.9% for anxiety). This is consistent with research findings where ethnic minorities were more at risk of negative psychological outcomes [6]. This pooled prevalence represented an increase in such conditions compared to another meta-analysis published in 2020 that showed them to be 15.9% for depression and 15.1% for anxiety. This increase in prevalence highlights the impact of COVID-19 on HCWs’ psychological well-being. Locally, studies have shown strikingly higher rates than international rates for depression (69%), anxiety (58.9%), stress (55.9%), and insomnia (37.3) [18]. Female HCWs are particularly more prone to suffering from psychological distress such as depression and anxiety [3,7,8,9,19].

Numerous studies have suggested possible reasons for such high rates of psychological distress among HCWs, which included increased workload, feeling isolated, reduced confidence in adopting safety procedures, fear of being infected, and lack of adequate protective equipment [20,21,22]. Therefore, continued investigations into the role of other relevant psychological variables, such as fear of COVID-19 and perceived vulnerability to disease, are necessary to understand the complexities underlying the emergence and continuation of symptoms of psychological distress. While high levels of fear are linked directly to anxiety, it appears unrelated to the extent to which an individual engages in protective behaviors [19]. This effect may be entirely different in the context of HCWs, where use of protective equipment is not only applied to decrease an unknown probability of encountering a potential source of infection but where there are confirmed cases of COVID-19.

The present study thus collected data from an HCW sample to investigate the relationship between fear of COVID-19, previous exposure to COVID-19, perceived vulnerability to disease and insomnia, and psychological factors including depression, anxiety, and stress. In light of the reviewed literature, it was hypothesized that HCWs might have high rates of depressive and anxiety symptoms as well as higher rates of insomnia and stress levels. Therefore, this study investigated the relationship between fear of COVID-19, previous exposure to COVID-19, perceived vulnerability to disease, sleep quality, and psychological distress among HCWs.

## 2. Materials and Methods

### 2.1. Participants

The Saudi Ministry of Health designated King Faisal Medical Complex (KFMC), which has more than 1400 HCWs, as the treatment provider for COVID-19 cases in Taif city. Only frontline HCWs (i.e., doctors and nurses) were included. The minimum required sample size for multiple regression with 11 predictors to achieve a statistical power of 95% to detect a small effects size of 0.20 under *p* = 0.05 was 136 participants. A cross-sectional study design was adopted for the purpose of this study. Invitations for voluntary participation were sent through the hospital intranet electronic mailing system. This was conducted via an open electronic survey, where a Google survey form was sent to all HCWs. As traditional convenience sampling methodology is known to be less generalizable and accurate than a homogeneous convenience sampling strategy, which can lead to estimation bias [23], a homogeneous sampling strategy was adopted in this study. This was due to the fact that HCWs are a homogeneous group that are different from other health professions.

### 2.2. Procedure

Consenting participants were asked to take an online anonymous survey investigating sociodemographic and background data along with different scales pertaining to fear of COVID-19, psychological aspects, anxiety, psychological distress. The data were collected within October–November of 2020. This study was approved by the institutional ethics committee of Taif University (IRB:HAO-02-T-105) and the Taif City Health Directorate (IRB:HAP-02-T-067-407) on 10 June 2020 and 13 September 2020, consecutively.

### 2.3. Measures

All questionnaires were presented in their English-language versions. The participants were HCWs who had either completed their training in an English-speaking environment, or English was an integral part of their education. Although two of the scales used in this study (i.e., Fear of COVID-19 and Depression and Anxiety Stress Scales) have previously been validated in Arabic [24,25], we used the English version as not all participants spoke Arabic. Therefore, the scales were administered in English, as all HCWs spoke English fluently. Less than 1% of the data were missing, not revealing any detectable pattern.

Fear of COVID-19 Scale (FCV-19S): The FCV-19S [26] presents seven items to be rated on a five-point Likert scale, ranging from 1 (strongly disagree) to 5 (strongly agree). Items are summed to calculate a total score, where a higher score represent higher levels of fear of COVID-19. The FCV-19S has robust psychometric properties and is typically interpreted as a unidimensional profile [26]. In the present sample, Cronbach’s alpha was 0.89. The average score for the items when using the scale with a Saudi Arabian sample was 2.42. There are currently no cut-off values available to classify respondents as expressing different levels of fear.

Depression Anxiety Stress Scale (DASS-21): Psychological distress was measured using the DASS-21 [27]. The instrument presents 21 statements on a four-point Likert scale (0 = never, 1 = sometimes, 2 = often, 3 = almost always), of which seven each relate to one of the subscales for depression, anxiety, and stress. Due to the fact of a questionnaire formatting error, item 6 (“I tended to over-react to situations”) was omitted, and the subscale score for stress was thus calculated with the six remaining subscale items. For all subscales, a higher score expressed a higher level of psychological distress. The DASS-21 has been used widely with generally acknowledged robust psychometric properties [28]. For the present sample, Cronbach’s alpha values of the depression, anxiety, and stress subscales were 0.86, 0.83, and 0.87, respectively. To enable comparison with the full 42-item cut-off scores, values for the subscales were multiplied by two. The following cut-off values were proposed [27] for the depression subscale: 0–9 for normal, 10–13 for mild, 14–20 for moderate, 21–27 for severe, and above 27 for extremely severe. For anxiety, these values were 0–7, 8–9, 10–14, 15–19, and above 19. For the stress subscale, the comparative cut-off values could not be used due to the fact that one item was excluded in the present analyses.

Pittsburgh Sleep Quality Index (PSQI): The PSQI [29] was used to assess sleep quality, where participants responded to a series of Likert-scale items with a variety of formats. Due to the fact that many of the participants were regularly called to night-shift work, not all subscales of the instrument could adequately be used to assess sleep quality. Instead of the PSQI’s seven components, only the following five were used: subjective sleep quality (Component 1), sleep latency (Component 2), sleep duration (Component 3), use of sleep medication (Component 6), and daytime dysfunction (Component 7). For all components, a higher score represented lower quality sleep. A total sleep quality score was also calculated as the sum of all these component scores. Cronbach’s alpha for the summary score consisting of these five items was unacceptably low at 0.42. Cronbach’s alpha where the item deleted revealed that sleep duration (Component 3) was an unreliable item. After deletion of that item, the Cronbach’s alpha for the remaining four items increased to 0.61. Given the different ways in which the scale’s total score was calculated, no comparable summary scores were available.

Perceived Vulnerability to Disease (PVD): The PVD [30], which has shown good psychometric properties, presents 15 items on a seven-point Likert scale ranging from 1 = “strongly disagree” to 7 = “strongly agree”. Six of the items were positively worded and thus rescored so that high scores on all items represented a high degree of perceived disease vulnerability. Items 2, 5, 6, 8, 10, 12, and 14 were then summed to calculate the subscale score called perceived infectability, and germ aversion was calculated as the sum of items 1, 3, 4, 7, 9, 11, 13, and 15. Cronbach’s alpha values for the two subscales were 0.52 and 0.54, respectively. No comparative mean values are available for this scale using samples in Saudi Arabia. In a recent study about fear of COVID-19 in South Africa, mean values for this scale were reported to be 28.7 (SD = 8.8) for perceived infectability and 42.8 (SD = 8.4) for germ aversion [31].

### 2.4. Data Analyses

All statistical analyses were conducted using the software package IBM SPSS v.27 (Armonk, NY, USA). All questions were compulsory; therefore, there were no missing values. Prior to conducting inferential statistics, the continuous variables were scrutinized for any deviations against the assumption of normality. Of the five components of the PSQI, Components 6 (use of sleep medication) and 7 (daytime dysfunction) had elevated skewness (2.04 and 1.41, respectively) and kurtosis (3.00 and 1.61, respectively). For that reason, only the total sleep scores were analyzed as opposed to the components. Pearson’s *r* correlation analysis explored the relationship between the continuous variables of interest, and a subsequent regression analysis explored predictors of psychological distress. Here, demographic factors were entered in the first block, followed by previous experience with COVID-19 in Block 2, and sleep quality, fear of COVID-19, and perceived disease vulnerability in Block 3.

## 3. Results

A total of two hundred and two HCWs participated in this study, which exceeded the calculated sample size of 136. The majority of the participants were female (71%, *n* = 144). Nurses represented the majority of the participants (67%, *n* = 136) while doctors represented a third of the participants (33%, *n* = 66). Approximately one-third of the participants held postgraduate degrees. The majority of participants were married (61%, *n* = 124). In terms of monthly income, the majority received less than USD 2666. Sociodemographic data and other characteristics of the sample are summarized in Table 1.

The average total summary score of the Fear of COVID-19 Scale was 18.54. In terms of item means, this was 2.65—a score slightly higher than that reported when using the scale in Saudi Arabia, albeit in the Arabic version [30].

For the depression subscale of the DASS-21, 59% (*n* = 119) of the participants were in the normal category, 14% (*n* = 29) in the category for mild depression, 19% (*n* = 39) in moderate depression, 3% (*n* = 6) in severe depression, and 5% (*n* = 9) in extremely severe depression. For anxiety, 50% (*n* = 100) were in the category normal, 6% (*n* = 12) mild, 24% (*n* = 48) moderate, 8% severe (*n* = 17), and 12% (*n* = 25) extremely severe. Table 2 shows these results by gender. For both depression (x2(4) = 9.80, *p* < 0.05) and anxiety (x2(4) = 18.27, *p* < 0.01), the gender differences were statistically significant. For the depression subscale, proportionally fewer males were in the normal category than females. For anxiety, this was reversed.

Table 3 shows a matrix of Pearson’s *r* correlation coefficients for the following variables: experience with COVID-19, sleep quality, fear of COVID-19, perceived infectability, germ aversion, depression, anxiety, and stress. Previous experience with actual COVID-19 cases was unrelated to any of the other variables. Fear of COVID-19 was weakly correlated with perceived infectability and germ aversion but moderately with depression, anxiety, and stress. Of the two perceived disease vulnerability subscales, only perceived infectability was weakly correlated with psychological distress, while germ aversion was not.

Subsequent multiple linear regression analyses explored the relationship between the variables of interest in more detail. Given the high correlation (>0.80) between the three subscales of the DASS-21 (Table 3) and the danger of collinearity, regression analyses were conducted separately for each of the three subscales as outcome variables, with none of the others added as predictor variables.

Detailed results are shown in Table 4. Of the demographic variables entered in Block 1, only gender was a significant predictor for depression, anxiety, and stress, and marital status for anxiety. Experience with COVID-19 was not a significant predictor for any of the three psychological distress subscales. The variance explained by Block 1 ranged from 0.11 to 0.18, and increased substantially with Block 3, ranging from 0.41 to 0.50. For all three psychological distress variables, Fear of COVID-19 as well as sleep quality were significant predictors, and the two subscales of the PVD (perceived infectability and germ aversion) were not significant.

## 4. Discussion

This study investigated the impact of COVID-19 on frontline HCWs’ psychological distress and insomnia among other variables. During the period of this study, there were 11,159 confirmed COVID-19 cases with more than 239 deaths in Taif city [32]. The sample size of 202 exceeded the required number of 105, and it was similar to previous research projects [3,33].

Our findings indicated that poor sleep quality was strongly associated with suffering from psychological distress. This is consistent with recent research findings among HCWs, where poor sleep quality mediated psychological distress [17]. According to this meta-analysis, the percentage for insomnia among HCWs was 39% [17]. The seriousness of insomnia was shown in one study where more than half of frontline HCWs suffered from moderate insomnia, while over a quarter of them suffered severe insomnia compared to non-frontline HCWs [34].

Fear of being infected is part of daily life for frontline HCWs in their work [35]. While normal fear helps people to adapt to threatening circumstances [36], excessive fear can be maladaptive [37]. Our study found that fear of COVID-19 was moderately associated with psychological distress. This is consistent with the international literature, as HCWs’ fear of acquiring COVID-19 infection was associated with psychological distress [17,38,39,40,41,42]. Some explanations provided in the literature for this heightened fear during pandemics included fear of uncertainty and acquiring the infection and transmitting it to others [40,43,44,45]. In the general population, fear of COVID-19 was similarly associated with high psychological distress [24,46].

Our study found fear of COVID-19 as a weak predictor for perceived vulnerability to disease. This was contrary to previous research that showed high correlation between the two variables [26], although another study conducted in Saudi Arabia found no association between perceived vulnerability to disease and stress [47]. One explanation for this may be that the current study took place a few months after the pandemic’s outbreak, which may have led to habituation in the fear response. For example, a study conducted in Germany found a reduction within six weeks, with fear of COVID-19 back to the level before lockdown [48].

Interestingly, experience in dealing with COVID-19 was neither a significant predictor for psychological distress nor was it correlated with Fear of COVID-19. This was consistent with the international literature, as clinicians interviewed at different time points in one study had lower levels of psychological distress compared to when the pandemic started [10]. This process of “normalization” was stated to be due to the fact of acquiring better knowledge and skills in dealing with the crisis, which was reported by 90% of the participants. Although they still had worries about their personal safety, all of the interviewed staff had adapted to the new way of working. This finding was supported by another study conducted in China that found that, over time, staff had adapted to dealing with the pandemic, i.e., “psychological adaptation” [49]. Therefore, it could be stated that previous experience in dealing with infectious diseases had no impact, as staff were more confident in dealing with the current crisis. Another reason could be that our study was conducted few months after the COVID-19 pandemic started, which gave staff enough time to adapt to the situation at hand.

In this study, participants’ gender was found to be a significant predictor of both depression and stress, where females had higher scores of depression. This is consistent with the findings of a number of local studies [3,8,9] that found higher rates of depression among female HCWs compared to their male counterparts. This fits well with the international literature, where it was found that females had higher rates of depression and stress [15,17]. Interestingly, our findings were not consistent with other local studies that found that females experienced higher rates of anxiety than males [3,7,50]. These local findings were consistent with the international literature that showed higher rates of anxiety among female HCWs [15,17]. One possible explanation for this could be that our study was conducted a few months into the pandemic, where psychological adaptation could have taken place [10,49]. The passage of time may have allowed more confidence to have been built through better knowledge, training, and graded exposure. This could have reduced the perceived danger associated with the pandemic thus lowering anxiety levels [10]. Moreover, past research showed that HCWs paid little attention to their psychological well-being during the peak of the COVID-19 pandemic and were less likely to seek help [51]. In addition, the persistence of depression and stress throughout the pandemic’s timeline could be explained by perceived helplessness and social isolation in the face of rising mortality [10].

Marital status was found to be a significant predictor of anxiety, confirming prior local studies. This was consistent with local and international research findings, where unmarried participants (i.e., single, divorced, and widowed) exhibited higher levels of psychological distress [3,13]. One explanation offered in the literature is fear and worries of infecting family members [10,13,52]. Dealing with the unfolding major effects of the pandemic might represent elevated uncertainty levels that are associated with higher anxiety levels [53], especially for those caring for a family.

In light of the above findings, there is a need for designing and implementing psychological support programs for HCWs with a preventive and therapeutic focus. These programs need to identify HCWs suffering from psychological distress in order to offer timely psychological support. In addition to the scales mentioned above, the literature offers quick depression screening methods such as Patient Health Questionnaire-9 (PHQ-9) which has been widely validated [54]. Finally, suicidal behavior being a serious consequence of elevated psychological distress should be screened for and managed accordingly. This could be performed using the Beck Scale for Suicidal Ideation, which also has good validity in assessing suicidal ideation [55].

### Strengths and Limitations

This study had a number of strengths that included using well-validated scales targeting relevant outcome measures among HCWs. However, the study had a number of limitations. For example, this study was conducted in one COVID center in one city in Saudi Arabia, which limits the findings’ generalizability. In addition, the cross-sectional design of this study does not infer causal associations, neither does it differentiate between pre-existing and current mental health problems. Moreover, selection bias could have taken place, as participation could have been influenced by certain staff’s individual concerns over COVID-19, thus limiting the sample’s representation. Furthermore, using a convenience sampling methodology could have led to selection bias. Another limitation is that suicidal ideation and behavior were not explored in this study, which could be an area for further research given its relationship with psychological distress especially during this pandemic [56]. Its importance lies in the fact that HCWs have higher rates of suicidal behavior than the general population, even before this pandemic began [57]. In addition, our study was conducted 8 months after the WHO declared COVID-19 a pandemic, which could have influenced the results as previous research has shown lower levels of psychological distress with the passage of time [49]. Finally, due to the fact of a formatting error, the stress subscale of the DASS-21 could not be interpreted in reference to known cut-off values

## 5. Conclusions

Psychological distress seems to be an ongoing issue for frontline HCWs dealing with the COVID-19 pandemic. Sleep quality and Fear of COVID-19 were strong and moderate predictors for all psychological distress, respectively. Female gender was strongly associated with depression and stress, while marital status was strongly associated with anxiety. Fear of COVID-19 was weakly associated with perceived infectability and germ aversion. Similarly, perceived infectability was weakly predictive of psychological distress. Finally, the level of experience and germ aversion were not predictors for psychological distress. From the above findings, our research findings lend support to calls for an urgent need to design and implement psychological programs to assist HCWs in dealing with the psychological sequelae of the ongoing pandemic.

## Figures and Tables

**Table 1:** Sociodemographic data and other characteristics of HCWs (*n* = 202). SD, standard deviation.

Variable	*n*	%	Mean	SD
Age			34.9	10.7
Gender				
Female	144	71		
Male	58	29		
Marital status				
Married	124	61		
Single (including divorced)	78	39		
Postgraduate education				
No	146	72		
Yes	56	28		
Employment				
Doctor	66	33		
Nurse	136	67		
Income				
USD < 2666.4	130	64		
USD 2666.4–4266.4	25	12		
USD > 4266.4	47	23		
Experience with COVID-19: “Have you dealt with previous versions of corona virus in any form? (e.g., have you worked in a hospital where they treated corona patients)”				
No	49	24		
Yes	153	76		
Fear of COVID-19			18.54	6.31
Sleep quality			8.10	3.11
Perceived infectability			26.68	6.02
Germ aversion			42.15	6.90
Depression			8.58	8.00
Anxiety			8.66	7.80
Stress			8.66	7.68

**Table 2:** Frequency of participants (by gender) in each of the depression and anxiety categories for the DASS-21.

	Depression		Anxiety	
	Male	Female	Male	Female
Normal	32	87	35	65
Mild	5	24	2	10
Moderate	14	25	5	43
Severe	1	5	3	14
Extremely severe	6	3	13	12

**Table 3:** Correlation coefficient matrix (Pearson’s *r*).

	Previous Experience with COVID-19	Fear of COVID-19	Perceived Infectability	Germ Aversion	Depression	Anxiety
Fear of COVID-19	0.07	-				
Perceived infectability	−0.07	0.22 **	-			
Germ aversion	−0.11	0.14 *	0.06	-		
Depression	−0.04	0.39 **	0.21 **	−0.02	-	
Anxiety	−0.05	0.44 **	0.21 **	0.02	0.83 **	-
Stress	0.00	0.41 **	0.23 **	−0.03	0.85 **	0.85 **

** *p* < 0.01; * *p* < 0.05 (two-tailed).

**Table 4:** Multiple linear regression. Results are shown separately for depression, anxiety, and stress as outcome variables. The demographic variables were entered as Block 1, experience with COVID-19 as Block 2, and independent variables as Block 3.

Step	R2	R2 Change	F (df1, df2)	Variable	Standardized β	*p*-Value
Depression						
1	0.18	0.18	6.67 (6, 180)			<0.01 **
				Age	−0.16	0.11
				Gender	−0.36	<0.01 **
				Marital status	0.13	0.13
				Postgraduate education	−0.01	0.92
				Employment type	0.17	0.23
				Income	−0.15	0.15
2	0.19	0.00	0.73 (1, 179)			0.39
				COVID-19 experience	−0.06	0.39
3	0.47	0.29	23.59 (4, 175)			<0.01 **
				Fear of COVID-19	0.32	<0.01 **
				Sleep quality	0.33	<0.01 **
				Perceived infectability	−0.00	0.98
				Germ aversion	0.03	0.60
Anxiety						
1	0.15	0.15	5.46 (6, 180)			<0.01 **
				Age	−0.15	0.15
				Gender	−0.18	0.05
				Marital status	0.20	<0.05 *
				Postgraduate education	−0.03	0.81
				Employment type	0.10	0.49
				Income	−0.08	0.47
2	0.16	0.00	0.56 (1, 179)			0.46
				COVID-19 experience	−0.05	0.46
3	0.50	0.35	30.49 (4, 175)			<0.01 **
				Fear of COVID-19	0.31	<0.01 **
				Sleep quality	0.40	<0.01 **
				Perceived infectability	−0.01	0.92
				Germ aversion	0.08	0.16
Stress						
1	0.11	0.11	3.61 (6, 180)			<0.01 **
				Age	−0.19	0.08
				Gender	−0.24	<0.05 *
				Marital status	0.08	0.37
				Postgraduate education	0.05	0.69
				Employment type	0.17	0.25
				Income	−0.09	0.41
2	0.11	0.00	0.06 (1, 179)			0.91
				COVID-19 experience	−0.02	0.81
3	0.41	0.30	22.32 (4, 175)			<0.01 **
				Fear of COVID-19	0.29	<0.01 **
				Sleep quality	0.36	<0.01 **
				Perceived infectability	0.04	0.56
				Germ aversion	0.04	0.49

Note: ** *p* < 0.01; * *p* < 0.05 (2-tailed). Abbreviation: R2, R-squared; df, degree of freedom.

## Data Availability

Not applicable.

## References

[1] Xiao H., Zhang Y., Kong D., Li S., Yang N. (2020). The Effects of Social Support on Sleep Quality of Medical Staff Treating Patients with Coronavirus Disease 2019 (COVID-19) in January and February 2020 in China. Med. Sci. Monit. Int. Med. J. Exp. Clin. Res..

[2] WHO Statement on the Second Meeting of the International Health Regulations (2005). Emergency Committee Regarding the Outbreak of Novel Coronavirus (2019-NCoV). https://www.who.int/news-room/detail/30-01-2020-statement-on-the-second-meeting-of-the-international-health-regulations-.

[3] Almater A.I., Tobaigy M.F., Younis A.S., Alaqeel M.K., Abouammoh M.A. (2020). Effect of 2019 Coronavirus Pandemic on Ophthalmologists Practicing in Saudi Arabia: A Psychological Health Assessment. Middle E. Afr. J. Ophthalmol..

[4] Li Y., Scherer N., Felix L., Kuper H. (2021). Prevalence of Depression, Anxiety and Post-Traumatic Stress Disorder in Health Care Workers during the COVID-19 Pandemic: A Systematic Review and Meta-Analysis. PLoS ONE.

[5] Yang L., Yin J., Wang D., Rahman A., Li X. (2021). Urgent Need to Develop Evidence-Based Self-Help Interventions for Mental Health of Healthcare Workers in COVID-19 Pandemic. Psychol. Med..

[6] Greenberg N. (2020). Mental Health of Health-Care Workers in the COVID-19 Era. Nat. Rev. Nephrol..

[7] Al Ammari M., Sultana K., Thomas A., Al Swaidan L., Al Harthi N. (2021). Mental Health Outcomes Amongst Health Care Workers During COVID 19 Pandemic in Saudi Arabia. Front. Psychiatry.

[8] AlAteeq D.A., Aljhani S., Althiyabi I., Majzoub S. (2020). Mental Health among Healthcare Providers during Coronavirus Disease (COVID-19) Outbreak in Saudi Arabia. J. Infect. Public Health.

[9] Alyami M., de Albuquerque J.V., Krägeloh C.U., Alyami H., Henning M.A. (2021). Effects of Fear of COVID-19 on Mental Well-Being and Quality of Life among Saudi Adults: A Path Analysis. Saudi J. Med. Med. Sci..

[10] Ardebili M.E., Naserbakht M., Bernstein C., Alazmani-Noodeh F., Hakimi H., Ranjbar H. (2020). Healthcare Providers Experience of Working during the COVID-19 Pandemic: A Qualitative Study. Am. J. Infect. Control.

[11] Chatterjee S.S., Bhattacharyya R., Bhattacharyya S., Gupta S., Das S., Banerjee B.B. (2020). Attitude, Practice, Behavior, and Mental Health Impact of COVID-19 on Doctors. Indian J. Psychiatry.

[12] Chew N.W.S., Lee G.K.H., Tan B.Y.Q., Jing M., Goh Y., Ngiam N.J.H., Yeo L.L.L., Ahmad A., Ahmed Khan F., Napolean Shanmugam G. (2020). A Multinational, Multicentre Study on the Psychological Outcomes and Associated Physical Symptoms amongst Healthcare Workers during COVID-19 Outbreak. Brain. Behav. Immun..

[13] Elbqry M.G., Elmansy F.M., Elsayed A.E., Mansour B., Tantawy A., Eldin M.B., Sayed H.H. (2021). Effect of COVID-19 Stressors on Healthcare Workers’ Performance and Attitude at Suez Canal University Hospitals. Middle E. Curr. Psychiatry.

[14] Hummel S., Oetjen N., Du J., Posenato E., de Almeida R.M.R., Losada R., Ribeiro O., Frisardi V., Hopper L., Rashid A. (2021). Mental Health Among Medical Professionals During the COVID-19 Pandemic in Eight European Countries: Cross-Sectional Survey Study. J. Med. Internet Res..

[15] Lai J., Ma S., Wang Y., Cai Z., Hu J., Wei N., Wu J., Du H., Chen T., Li R. (2020). Factors Associated With Mental Health Outcomes Among Health Care Workers Exposed to Coronavirus Disease 2019. JAMA Netw. Open.

[16] Mohd Fauzi M.F., Mohd Yusoff H., Muhamad Robat R., Mat Saruan N.A., Ismail K.I., Mohd Haris A.F. (2020). Doctors’ Mental Health in the Midst of COVID-19 Pandemic: The Roles of Work Demands and Recovery Experiences. Int. J. Environ. Res. Public Health.

[17] Pappa S., Ntella V., Giannakas T., Giannakoulis V.G., Papoutsi E., Katsaounou P. (2020). Prevalence of Depression, Anxiety, and Insomnia among Healthcare Workers during the COVID-19 Pandemic: A Systematic Review and Meta-Analysis. Brain. Behav. Immun..

[18] Arafa A., Mohammed Z., Mahmoud O., Elshazley M., Ewis A. (2021). Depressed, Anxious, and Stressed: What Have Healthcare Workers on the Frontlines in Egypt and Saudi Arabia Experienced during the COVID-19 Pandemic?. J. Affect. Disord..

[19] Lim X.Y., Yap A.C., Mahendran R., Yu J. (2021). The Interplay between Anxiety, Fear, Protective Behaviors, Compassion, and Resilience among Older Adults during a COVID-19 Lockdown: A Structural Equation Modeling Study. Transl. Behav. Med..

[20] Magnavita N., Soave P.M., Antonelli M. (2021). Prolonged Stress Causes Depression in Frontline Workers Facing the COVID-19 Pandemic—A Repeated Cross-Sectional Study in a COVID-19 Hub-Hospital in Central Italy. Int. J. Environ. Res. Public. Health.

[21] De Kock J.H., Latham H.A., Leslie S.J., Grindle M., Munoz S.-A., Ellis L., Polson R., O’Malley C.M. (2021). A Rapid Review of the Impact of COVID-19 on the Mental Health of Healthcare Workers: Implications for Supporting Psychological Well-Being. BMC Public Health.

[22] Magnavita N., Tripepi G., Di Prinzio R.R. (2020). Symptoms in Health Care Workers during the COVID-19 Epidemic. A Cross-Sectional Survey. Int. J. Environ. Res. Public. Health.

[23] Jager J., Putnick D.L., Bornstein M.H. (2017). More than Just Convenient: The Scientific Merits of Homogeneous Convenience Samples. Monogr. Soc. Res. Child Dev..

[24] Alyami M., Henning M., Krägeloh C.U., Alyami H. (2020). Psychometric Evaluation of the Arabic Version of the Fear of COVID-19 Scale. Int. J. Ment. Health Addict..

[25] Moussa M.T., Lovibond P., Laube R., Megahead H.A. (2017). Psychometric Properties of an Arabic Version of the Depression Anxiety Stress Scales (DASS). Res. Soc. Work Pract..

[26] Ahorsu D.K., Lin C.-Y., Imani V., Saffari M., Griffiths M.D., Pakpour A.H. (2020). The Fear of COVID-19 Scale: Development and Initial Validation. Int. J. Ment. Health Addict..

[27] Lovibond S.H., Lovibond P.F. (1995). The structure of negative emotional states: Comparison of the Depression Anxiety Stress Scales (DASS) with the Beck Depression and Anxiety Inventories. Behav. Res. Ther..

[28] Medvedev O.N., Krägeloh C.U., Titkova E.A., Siegert R.J. (2020). Rasch Analysis and Ordinal-to-Interval Conversion Tables for the Depression, Anxiety and Stress Scale. J. Health Psychol..

[29] Buysse D.J., Reynolds C.F., Monk T.H., Berman S.R., Kupfer D.J. (1989). The Pittsburgh Sleep Quality Index: A New Instrument for Psychiatric Practice and Research. Psychiatry Res..

[30] Duncan L., Schaller M., Park J. (2009). Perceived Vulnerability to Disease: Development and Validation of a 15-Item Self-Report Instrument. Pers. Individ. Differ..

[31] Padmanabhanunni A., Pretorius T. (2021). “I Teach, Therefore I Am”: The Serial Relationship between Perceived Vulnerability to Disease, Fear of COVID-19, Teacher Identification and Teacher Satisfaction. Int. J. Environ. Res. Public. Health.

[32] COVID 19 Dashboard: Saudi Arabia. https://covid19.moh.gov.sa/.

[33] Al-Humadi S., Bronson B., Muhlrad S., Paulus M., Hong H., Cáceda R. (2021). Depression, Suicidal Thoughts, and Burnout Among Physicians During the COVID-19 Pandemic: A Survey-Based Cross-Sectional Study. Acad. Psychiatry.

[34] Wu K., Wei X. (2020). Analysis of Psychological and Sleep Status and Exercise Rehabilitation of Front-Line Clinical Staff in the Fight Against COVID-19 in China. Med. Sci. Monit. Basic Res..

[35] Cawcutt K.A., Starlin R., Rupp M.E. (2020). Fighting Fear in Healthcare Workers during the COVID-19 Pandemic. Infect. Control Hosp. Epidemiol..

[36] Steimer T. (2002). The Biology of Fear- and Anxiety-Related Behaviors. Dialogues Clin. Neurosci..

[37] García-Reyna B., Castillo-García G.D., Barbosa-Camacho F.J., Cervantes-Cardona G.A., Cervantes-Pérez E., Torres-Mendoza B.M., Fuentes-Orozco C., Pintor-Belmontes K.J., Guzmán-Ramírez B.G., Hernández-Bernal A. (2020). Fear of COVID-19 Scale for Hospital Staff in Regional Hospitals in Mexico: A Brief Report. Int. J. Ment. Health Addict..

[38] Abdelghani M., El-Gohary H.M., Fouad E., Hassan M.S. (2020). Addressing the Relationship between Perceived Fear of COVID-19 Virus Infection and Emergence of Burnout Symptoms in a Sample of Egyptian Physicians during COVID-19 Pandemic: A Cross-Sectional Study. Middle E. Curr. Psychiatry.

[39] Andrade E.F., Pereira L.J., de Oliveira A.P.L., Orlando D.R., Alves D.A.G., de Sales Guilarducci J., Castelo P.M. (2022). Perceived Fear of COVID-19 Infection According to Sex, Age and Occupational Risk Using the Brazilian Version of the Fear of COVID-19 Scale. Death Stud..

[40] Cabarkapa S., Nadjidai S.E., Murgier J., Ng C.H. (2020). The Psychological Impact of COVID-19 and Other Viral Epidemics on Frontline Healthcare Workers and Ways to Address It: A Rapid Systematic Review. Brain Behav. Immun. Health.

[41] Hawari F.I., Obeidat N.A., Dodin Y.I., Albtoosh A.S., Manasrah R.M., Alaqeel I.O., Mansour A.H. (2021). The Inevitability of Covid-19 Related Distress among Healthcare Workers: Findings from a Low Caseload Country under Lockdown. PLoS ONE.

[42] Hu D., Kong Y., Li W., Han Q., Zhang X., Zhu L.X., Wan S.W., Liu Z., Shen Q., Yang J. (2020). Frontline Nurses’ Burnout, Anxiety, Depression, and Fear Statuses and Their Associated Factors during the COVID-19 Outbreak in Wuhan, China: A Large-Scale Cross-Sectional Study. EClinicalMedicine.

[43] Maunder R., Hunter J., Vincent L., Bennett J., Peladeau N., Leszcz M., Sadavoy J., Verhaeghe L.M., Steinberg R., Mazzulli T. (2003). The Immediate Psychological and Occupational Impact of the 2003 SARS Outbreak in a Teaching Hospital. CMAJ Can. Med. Assoc. J..

[44] Shacham M., Hamama-Raz Y., Kolerman R., Mijiritsky O., Ben-Ezra M., Mijiritsky E. (2020). COVID-19 Factors and Psychological Factors Associated with Elevated Psychological Distress among Dentists and Dental Hygienists in Israel. Int. J. Environ. Res. Public. Health.

[45] Urooj U., Ansari A., Siraj A., Khan S., Tariq H. (2020). Expectations, Fears and Perceptions of Doctors during Covid-19 Pandemic. Pak. J. Med. Sci..

[46] Soraci P., Ferrari A., Abbiati F.A., Del Fante E., De Pace R., Urso A., Griffiths M.D. (2020). Validation and Psychometric Evaluation of the Italian Version of the Fear of COVID-19 Scale. Int. J. Ment. Health Addict..

[47] Pasay-an E. (2020). Exploring the Vulnerability of Frontline Nurses to COVID-19 and Its Impact on Perceived Stress. J. Taibah Univ. Med. Sci..

[48] Hetkamp M., Schweda A., Bäuerle A., Weismüller B., Kohler H., Musche V., Dörrie N., Schöbel C., Teufel M., Skoda E.-M. (2020). Sleep Disturbances, Fear, and Generalized Anxiety during the COVID-19 Shut down Phase in Germany: Relation to Infection Rates, Deaths, and German Stock Index DAX. Sleep Med..

[49] Zhang Y., Wei L., Li H., Pan Y., Wang J., Li Q., Wu Q., Wei H. (2020). The Psychological Change Process of Frontline Nurses Caring for Patients with COVID-19 during Its Outbreak. Issues Ment. Health Nurs..

[50] Alzaid E.H., Alsaad S.S., Alshakhis N., Albagshi D., Albesher R., Aloqaili M. (2020). Prevalence of COVID-19-Related Anxiety among Healthcare Workers: A Cross-Sectional Study. J. Fam. Med. Prim. Care.

[51] Chen Q., Liang M., Li Y., Guo J., Fei D., Wang L., He L., Sheng C., Cai Y., Li X. (2020). Mental Health Care for Medical Staff in China during the COVID-19 Outbreak. Lancet Psychiatry.

[52] Mekonen E., Shetie B., Muluneh N. (2021). The Psychological Impact of COVID-19 Outbreak on Nurses Working in the Northwest of Amhara Regional State Referral Hospitals, Northwest Ethiopia. Psychol. Res. Behav. Manag..

[53] Grupe D.W., Nitschke J.B. (2013). Uncertainty and Anticipation in Anxiety. Nat. Rev. Neurosci..

[54] Costantini L., Pasquarella C., Odone A., Colucci M.E., Costanza A., Serafini G., Aguglia A., Belvederi Murri M., Brakoulias V., Amore M. (2021). Screening for Depression in Primary Care with Patient Health Questionnaire-9 (PHQ-9): A Systematic Review. J. Affect. Disord..

[55] Beck A.T., Brown G.K., Steer R.A. (1997). Psychometric Characteristics of the Scale for Suicide Ideation with Psychiatric Outpatients. Behav. Res. Ther..

[56] Mohsin S.F., Agwan M.A., Shaikh S., Alsuwaydani Z.A., AlSuwaydani S.A. (2021). COVID-19: Fear and Anxiety among Healthcare Workers in Saudi Arabia. A Cross-Sectional Study. Inq. J. Health Care Organ. Provis. Financ..

[57] Davidson J.E., Proudfoot J., Lee K., Terterian G., Zisook S. (2020). A Longitudinal Analysis of Nurse Suicide in the United States (2005–2016) With Recommendations for Action. Worldviews Evid. Based Nurs..

